# Cardiac 31 P MRS analysis development: Improved fitting of 2, 3 DPG

**DOI:** 10.1186/1532-429X-15-S1-P219

**Published:** 2013-01-30

**Authors:** Sairia Dass, Damian Tyler, Stefan Neubauer, Kieran Clarke, Lowri E Cochlin

**Affiliations:** 1 OCMR, John Radcliffe Hospital, Oxford, UK; 2 Department of Physiology, Anatomy and Genetics, Oxford University, Oxford, UK

## Background

31P MRS is an important tool for the measurement of in vivo energetics via the ratio PCr/ATP. Low signal-to-noise (31P molar receptivity is 1/14 that of 1H) coupled with low concentration of high-energy phosphates can result in high measurement variability. 31P MRS of human heart often contains signals originating from blood i.e. 2,3DPG and ATP. Measurement of myocardial PCr/ATP thus requires subtraction of blood derived ATP signal. The aim of this work was to improve blood correction by applying prior knowledge (PK) of molecular spectral parameters to the 2,3DPG signal. We demonstrate that accurate spectral fitting of 2,3DPG prevents proliferation of fitting errors through blood correction, resulting in improved accuracy of PCr/ATP measurement.

## Methods

A review of the literature for spectral information about 2,3DPG was employed to guide changes to the existing PK for 2,3DPG, Table 1. Sixteen 31 P spectra were simulated in jMRUI, PCr/ATP ratio fixed at 2.00 with 20% and 40% noise added. Forty eight datasets from 8 HCM, 8 DCM and 8 normal controls were analyzed. The original and the new 2,3DPG fitting PKs were applied to the each simulated and patient dataset.

**Table 1:** A summary of the differences between the original and new prior knowledge for 2, 3 DPG.

	PARAMETER	OLD PK	NEW PK
2 DPG	Amplitude estimate	estimate	Fixed 3 DPGx0.8

	Relative Phase	FIxed 0	Fixed 0

	Line Width	estimate	20-100

	Frequency	Soft constraint	5.01-6.01

	Shape	Lorentizian	Lorentizian

	PARAMETER	OLD PK	NEW PK

3 DPG	Amplitude estimate	estimate	estimate

	Relative Phase	FIxed 0	FIxed 0

	Line Width	estimate	2 DPGx1

	Frequency	Soft constraint	6.02-6.81

	Shape	Lorentizian	Lorentizian

## Results

### Simulated Data

In the data with 20% noise, there were no differences in the mean and standard deviations (SD) of the PCr/ATP ratios with the original ( 2.02±0.13) and new 2,3 DPG fit ( 2.03±0.12, P=0.3). However, with 40% noise, both the mean and SD of the PCr/ATP ratios using the original 2,3DPG fit, were significantly higher than with using the new 2,3DPG fit (original fit: PCr/ATP 2.77±0.99; new fit: PCr/ATP 2.33±0.46, p<0.05). The mean difference of the measured PCr/ATP ratio and the actual ratio of 2.00 was 0.77±0.99 for the original fit and 0.33±0.46 for the new fit, P=0.02.

### Patient Data

**Figure 1:**
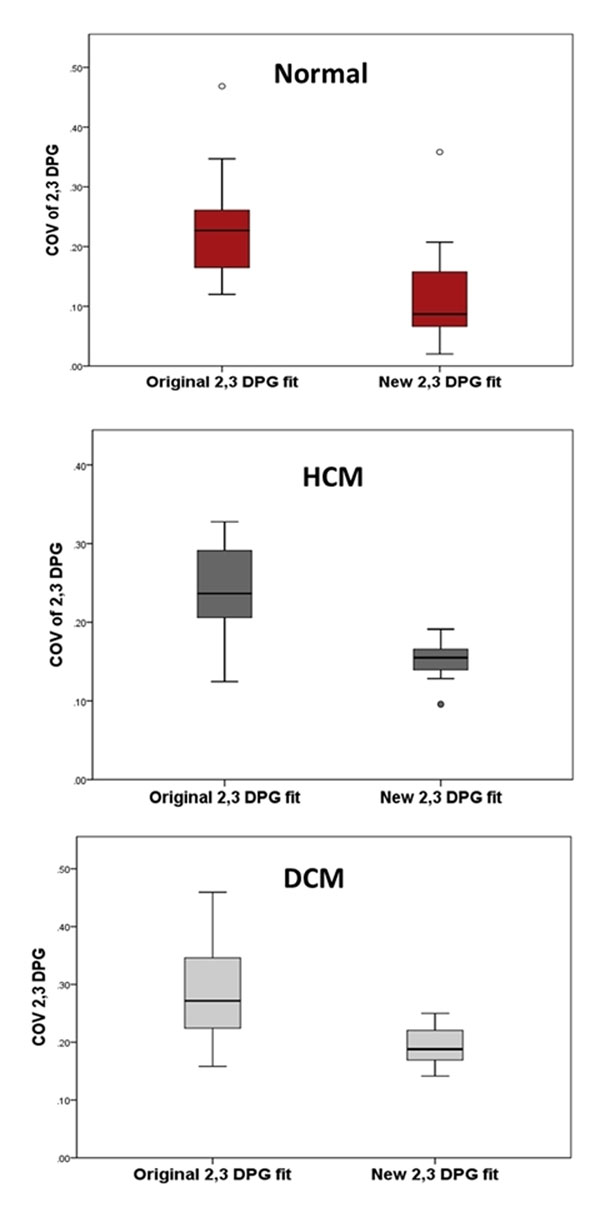
Box plot of the distribution of the coefficient of variance of 2, 3 DPG measurements for normal volunteers, HCM and DCM cohorts using the original and new 2, 3 DPG fits. Center of box represents median, with interquartile limits, whiskers extend to the maximum and minimum ratios.

There was no difference in the mean PCr/ATP with the different PKs (original fit: HCM 1.96±0.53, DCM 1.47±0.28, normal 2.26±0.32; new fit: HCM 1.80±0.33, DCM 1.51±0.20, normal 2.22±0.28, P>0.1). However, with the new PK, there was a substantial improvement in the coefficient of variance of the 2,3DPG fit in all groups (original fit vs. new fit: HCM 24% vs 15% P<0.005; DCM 22% vs 12% P<0.005; normal controls 23% vs 15% P<0.0005, Figure 1). This translated into a reduction in the SD of the PCr/ATP measurements in all 3 groups. In HCM and DCM, based on this pilot data, in order to detect a 10% change in PCr/ATP with an intervention (α 0.05, power 80%), the number of subjects needed with the new 2,3DPG PK is reduced from 59 to 27 in HCM and 28 to 14 in DCM.

## Conclusions

31P MRS is a valuable tool for assessment of myocardial metabolism.. This work developed an improved fit for 2,3DPG hence minimising propagation of errors to the final PCr/ATP calculation. The quality of this outcome is reflected in the power calculations which illustrate an almost 50% reduction of sample size required to statistically demonstrate a change in PCr/ATP with an intervention in HCM and DCM.

## Funding

British heart foundation

